# The impact of prolonged walking on fasting plasma glucose in type 2 diabetes: A randomised controlled crossover study

**DOI:** 10.1111/dme.15468

**Published:** 2024-11-09

**Authors:** Anxious J. Niwaha, Beverley M. Shields, Lauren R. Rodgers, Andrew T. Hattersley, Robert C. Andrews, Moffat J. Nyirenda, Angus G. Jones

**Affiliations:** ^1^ Institute of Biomedical and Clinical Science, College of Medicine and Health University of Exeter Medical School Exeter UK; ^2^ Medical Research Council/Uganda Virus Research Institute and LSHTM Uganda Research Unit Entebbe Uganda; ^3^ Institute of Health Research, College of Medicine and Health University of Exeter Exeter UK; ^4^ Department of Non‐communicable Diseases Epidemiology London School of Hygiene and Tropical Medicine (LSHTM) London UK

**Keywords:** exercise, fasting glucose, randomised crossover trial, type 2 diabetes

## Abstract

**Aims:**

In many low‐income countries, fasting glucose is the primary measure for monitoring glycaemic control. Many patients in these countries walk long distances to the clinic, but the impact of walking on fasting glucose in type 2 diabetes is unknown. We aimed to determine the impact of walking on fasting glucose in people with type 2 diabetes.

**Methods:**

In a randomised crossover trial, the change in glucose from baseline in the fasting state was compared between walking on a treadmill at a predetermined speed of 4.5 km/h for 1 h and not walking (resting) in people with type 2 diabetes.

**Results:**

In all, 45 participants were enrolled and all completed both visits; 21/45 (46.7%) were women, and the median age was 51. Glucose during and after walking was similar to glucose while at rest; the glucose difference (walking minus rest) was −0.15 (95% CI: −0.55, 0.26) and −0.10 (95% CI: −0.50, 0.31) mmol/L at 1 and 2 h, respectively, *p* > 0.4 for both.

**Conclusions:**

Fasting plasma glucose is not meaningfully affected by prolonged walking in participants with type 2 diabetes; therefore, the reliability of fasting glucose for monitoring glycaemic burden is unlikely to be altered in patients who walk to the clinic.

**What's new?**

**What is already known?**

- Fasting glucose is widely used to assess glycaemic control in people living with diabetes in low‐income countries.
- Many patients in these countries walk long distances to the clinic, but little is known about the impact of walking on fasting glucose in people living with diabetes.

**What this study has found?**

- Fasting plasma glucose is not meaningfully affected by prolonged walking in participants with type 2 diabetes.

**What are the implications of the study?**

- The reliability of fasting glucose for monitoring glycaemic burden is unlikely to be altered in patients who walk to the clinic.

## 1 INTRODUCTION

HbA1c and home capillary or subcutaneous glucose monitoring are the primary measures used to monitor glycaemic control and guide treatment titration in diabetes in high‐income countries. However, these approaches are often unavailable or unaffordable^1^, ^2^ for many of the 432.7 million people with diabetes who live in low‐ or middle‐income countries.^3^ In this setting, international organisations recommend the use of fasting glucose to monitor glycaemic control and titrate glucose‐lowering therapy, and this remains the primary means of glucose monitoring for a substantial proportion of those living with diabetes worldwide.^4^

In many low‐ and middle‐income countries, diabetes clinics are operated at regional and district hospitals, and access to transport services may be unreliable or unaffordable. Therefore, many patients will walk long distances to attend diabetes clinics.^2^ While exercise such as walking results in increased glucose uptake and utilisation by the exercising muscles,^5^ it remains unclear whether the fasting plasma glucose measure is affected by a single bout of exercise such as walking in individuals with type 2 diabetes (T2D). We, therefore, aimed to determine the impact of walking on fasting glucose in people living with T2D.

## 2 SUBJECTS, MATERIALS, AND METHODS

### 2.1 Study design and patients

The study was a multicentre randomised two‐period crossover design to test the impact of walking on a treadmill for 1 h compared to not‐walking on fasting glucose changes in participants with type 2 diabetes. The Uganda Virus Research Institute (UVRI) Institutional Review Board and the Uganda National Council of Science and Technology (UNCST) approved the study, and all participants provided informed consent before participating.

We enrolled 45 non‐insulin‐treated participants with T2D attending diabetes outpatient clinics at one rural and one urban hospital in Uganda. Exclusion criteria included pregnancy, acute illness, and clinical need to immediately increase their glucose‐lowering medication.

**FIGURE 1:**
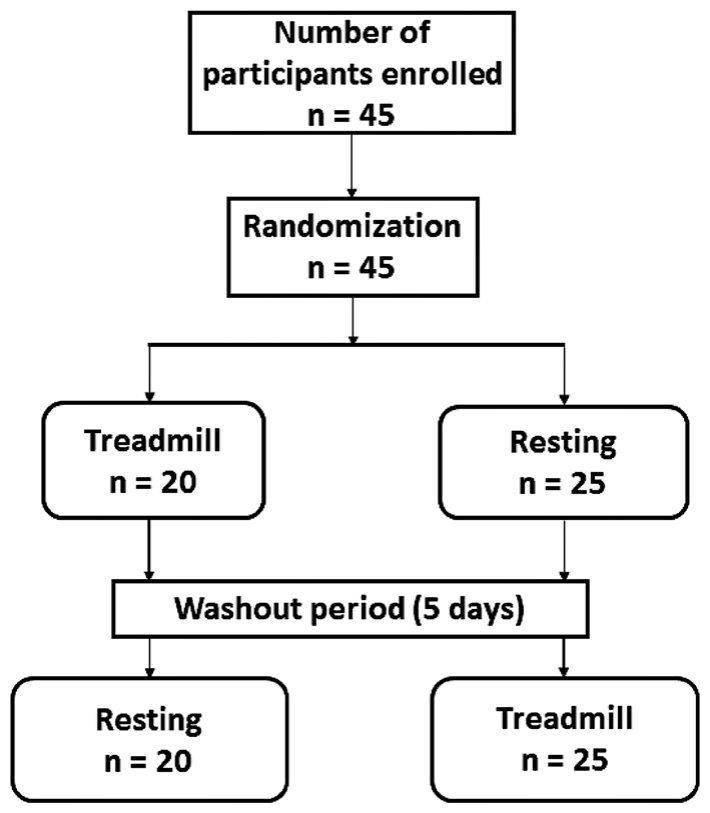
Participant flow chart. Participants attended two study visits: Treadmill visit; participant walks on a treadmill at a speed of 4.5 km/h for 60 min and then rests for the next 2 h, resting visit; participant rests for 3 h with minimal activity. The primary outcome was a change in fasting glucose between the walking and rest visit at 1 and 2‐h post‐treatment.

The study was registered in the Pan African Clinical Trial Registry (https://pactr.samrc.ac.za/) (PACTR202009486614518). The overview of the study design is presented in Figure 1.

### 2.2 Study visits and procedures

Participants attended two study visits: an exercise visit and a resting visit, in random order, as assigned by an independent person using computer‐generated random numbers. For both visits, participants fasted (>8 h) before 9 AM. Activity pre‐visit was minimised by funding visit transport and providing specific participant advice to minimise activity, and participants withheld their glucose‐lowering medications on the morning of the visits. There was a 5‐day washout period between visits. Blinding was not possible for this study.

### 2.3 Exercise visit

Participants exercised at a moderate walking pace of 3 mph (4.5 km/h) on a treadmill for 1 h. This speed was chosen to represent average walking speed.^6^ Blood samples were taken for laboratory glucose measurement at 0, 30 and 60 min, and every 30 min for a further 2 h in the post‐exercise phase (resting) (3 h total monitoring).

### 2.4 Rest visit

Participants rested (while seated) for 3 h. Samples were taken for laboratory glucose measurement at 0, 30 and 60, 90, 120, 150 and 180 min.

### 2.5 Primary outcome

The pre‐specified co‐primary outcomes were change in fasting glucose between the walking and rest visits at 60 and 120 min post‐commencing the intervention.

### 2.6 Laboratory analysis

Blood samples for glucose measurement were collected in a vacutainer with sodium fluoride (NaF), centrifuged and separated into two cryovials (aliquots) immediately and kept in an icebox at 4–8°C before being transported to the central laboratory for immediate testing (within 8 h of collection). Plasma glucose was measured by the glucokinase method on a Roche cobas 6000 analyser (Hitachi High Technologies Corporation, Tokyo, Japan) at the Central Biochemistry and Clinical Diagnostic Laboratory Services (CDLS) laboratory at the MRC/UVRI and LSHTM Research Unit Entebbe Uganda.

### 2.7 Statistics

The study aimed for a sample size of at least 38 with the primary outcome, which would have 80% power to detect a 1 mmol/L (0.47 standard deviation) difference in fasting glucose change at 1 and 2 h between the walking and rest visit with an alpha of 0.05.

We assessed the carryover effect (which occurs when the effect of the first treatment continues until the next period and alters outcomes during the subsequent treatment)^7^ and the period effect (the effect of the same treatment received at two different periods is different for each period)^7^ using mixed‐effects models with 60‐min glucose as the outcome, intervention group, period, and intervention group × period interaction as independent variables, and participant ID as the random effect. Without excluding any data, we compared the difference in glucose change from baseline between resting and exercising visits at 60 and 120 min from the start, using paired *t*‐tests. The overall impact of walking (across all the time points 30–180 min) was assessed using mixed‐effect models adjusted for baseline (time 0) glucose with time points as fixed effects and patients as random effects. We also examined whether the exercise intervention explained further variability in glucose over the total duration using the likelihood ratio test to compare with the rest intervention model.

## 3 RESULTS

All 45 recruited participants completed both study visits (Figure 1). The overall characteristics of study participants and by sequence are shown in Table 1 and Supplementary Table S1, respectively. There was no evidence of period effect (*p* = 0.29) or carryover effect (*p* = 0.56).

**TABLE 1:** Baseline characteristics (*n* = 45).

Variables	Median (IQR), *n* (%)
	Overall
Age	51 (46, 56)
Women	21 (46.7)
Diabetes duration, years	4 (2.0, 7.0)
Treatment	
Metformin (±diet only)^a^	07 (15.6)
SU (±metformin)^b^	35 (77.8)
Other diabetes drugs^c^	03 (06.6)
BMI, kg/m^2^	26.7 (24.0, 29.7)
Fasting glucose, mmol/L	7.9 (5.5, 10.0)
HbA1c, mmol/mol	66 (46, 82)
HbA1c, %	8.2 (6.4, 9.6)
C‐peptide, pmol/L	1310 (878, 2030)

*Note*: Categorical data are presented as frequency (%), continuous data as median (IQR).

Abbreviation: BMI, body mass index.

^a^ Metformin and diet only (only 1 patient was on diet alone without pharmacological treatment).

^b^ Sulphonylurea with or without metformin.

^c^ Metformin with thiazolidinedione or dipeptidyl peptidase‐4 (DPP‐4) inhibitor.

### 3.1 Walking was not associated with a change in fasting glucose at the end of the exercise

**FIGURE 2:**
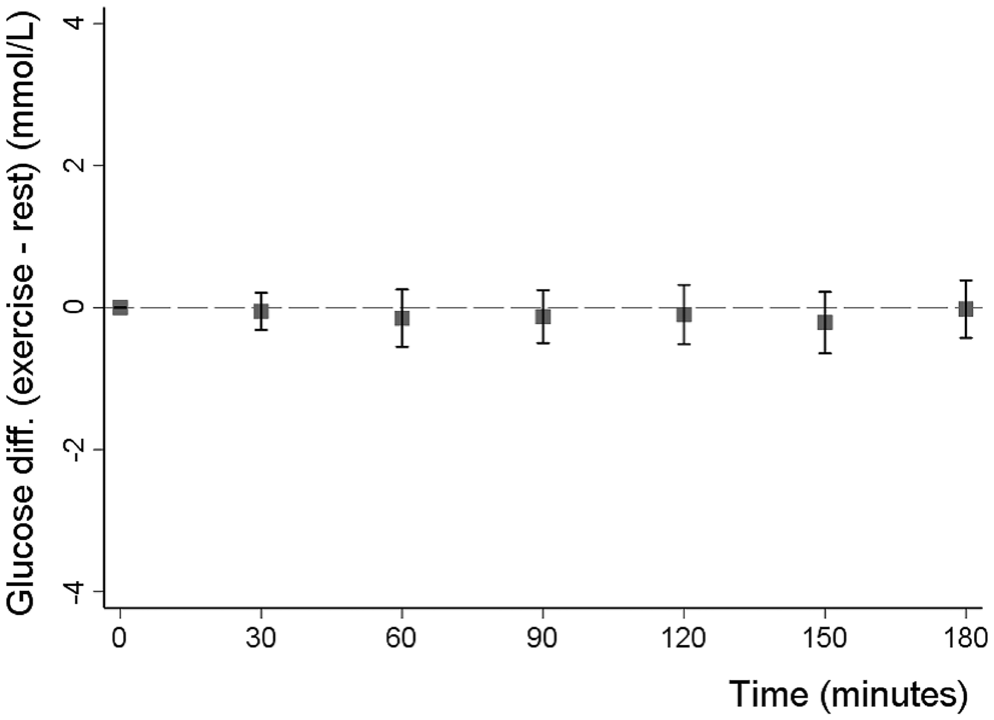
Mean difference (with 95% CIs) in glucose change from baseline between the exercise (walking) and resting visits. Y‐axis shows the difference in glucose change from baseline between the exercise and rest visits (exercise minus rest). The X‐axis shows time in minutes from baseline (0 min) up to 180 min from the start of the visits.

**FIGURE 3:**
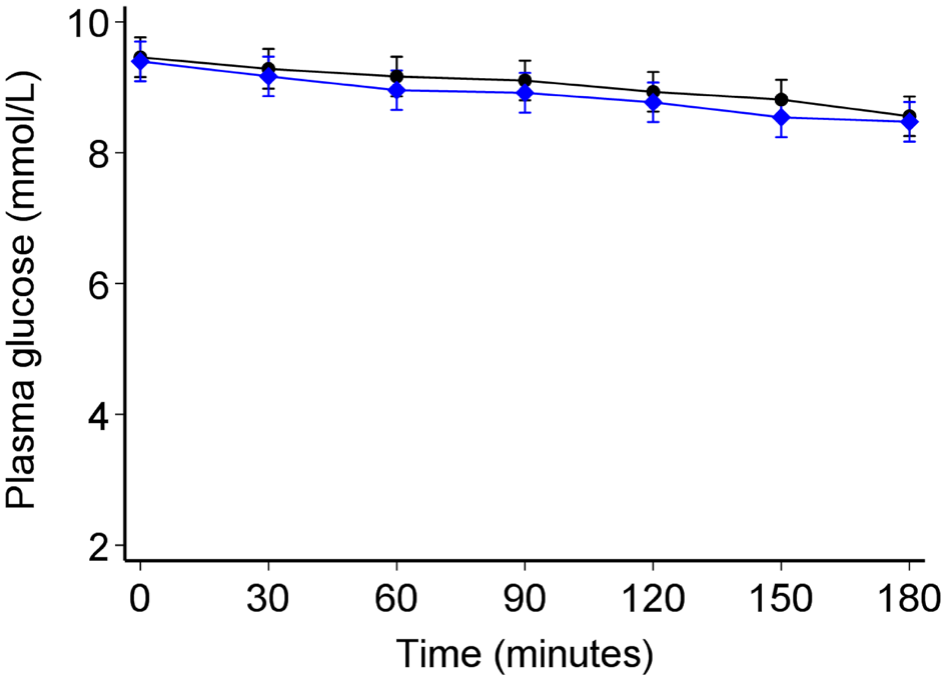
Glucose trends between the exercise (blue diamonds) and resting visit (black circles) after adjusting for the baseline differences between the two visits. The error bars denote 95% confidence intervals.

Walking for 1 h was not associated with changes in fasting glucose after 60 min of exercise or after an additional hour of rest. Compared to the resting (control visit) glucose change from baseline (pre‐intervention) with exercise was −0.15 (95% CI: −0.55, 0.26) mmol/L (*p* = 0.48) and −0.10 (95% CI: −0.50, 0.31) mmol/L (*p* = 0.64) at 60 and 120 min, respectively (Figure 2). The glucose difference was similar across all other post‐baseline time points (Figure 2). The absolute values for exercise and rest visits separately are shown in Figure 3.

#### 3.1.1 Walking was not associated with differences in overall post baseline glycaemia

When assessing all time points using a mixed‐effects model, there was no difference in glucose between visits (*p* = 0.67) over the 3 h post‐baseline (Figure 3). The addition of exercise into the model did not explain further variability in glucose [Likelihood‐ratio (LR) test *χ* ^2^ = 9.0, *p* = 0.25].

## 4 DISCUSSION

Our findings demonstrate that in people with type 2 diabetes, fasting glucose is not meaningfully altered by 1 h of walking.

There has been limited study of the effects of single bouts of exercise on fasting glucose in type 2 diabetes, with a recent systematic review identifying only 1 study that examined exercise in the fasting state with an appropriate comparison.^8^ Karstoft et al. examined the impact of continuous walking and interval walking for 1 h (60 min) in a crossover study of 10 participants and found no impact on fasting plasma glucose in comparison to the resting state.^9^ Consistent with our finding, studies assessing the overall effect of exercise (of any type) on glucose measured by continuous glucose monitoring (CGM) in those living with type 2 diabetes have also shown no acute effect on fasting glucose.^8^, ^10^ In contrast, previous studies have shown clearly that postprandial glucose is substantially impacted by a single bout of exercise.^9^, ^11^, ^12^

Strengths of our study in comparison to previous work include that we have studied a far larger population using a randomised cross‐over design and measured both pre‐ and post‐exercise glucose, and therefore we were able to control for baseline difference in the exercise and non‐exercise (control) arms.

The stable fasting glucose in the walking (exercising) visit suggests that short‐term moderate exercise does not affect fasting glucose, despite the known insulin‐independent effects of exercise on muscle glucose uptake.^13^ The mechanisms underlying this finding are unclear but may involve counter‐regulatory processes that favour fasting glucose stabilisation through glycogenolysis and increased mobilisation of alternative fuel sources such as free fatty acids (FFAs) from triglycerides in the adipose tissue.^9^, ^14^, ^15^

These findings show that the common occurrence of patients walking a long distance to the clinic is unlikely to alter fasting glucose, even in those with poor glycaemic control, and therefore should not affect test interpretation. Previous research indicates that a significant number of patients in Uganda and other SSA countries walk long distances to access their diabetes clinics, with over 70% of Ugandans living within a 1‐h walking distance to the nearest health centre.^2^, ^16^ In our study, the median time taken by participants to reach the diabetes clinic was 60 (IQR: 30, 90) minutes. Thus, the trial's design, which included a one‐hour treadmill walking session at a speed of 4.5 km/h, reflects real‐life conditions. These findings may also have implications for those undertaking moderate‐intensity exercise in the fasting state who are treated with sulfonylurea therapy, with recent guidance recommending carbohydrate pre‐exercise in those using SUs (insulin secretagogues) regardless of fasting state.^15^ A large proportion (44.4%) of our study participants received long‐acting first‐generation sulfonylurea treatment (glibenclamide), but did not experience a decline in glucose during exercise. This suggests that moderate exercise is safe even on SUs. However, this should be interpreted with the important caveats that (1) glucose lowering treatment was withheld on the morning of the study visits and (2) the distance chosen in the experiment was based on pragmatism and clinical experience, and, therefore, the findings may not apply to cases where patients walk longer distances.

In conclusion, fasting plasma glucose is not meaningfully affected by prolonged walking in participants with type 2 diabetes; therefore, the reliability of fasting glucose for monitoring glycaemic burden is unlikely to be altered in patients who walk to the clinic.

## AUTHOR CONTRIBUTIONS

A. J. N., B. S., A. T. H., M. J. N. and A. G. J. conceptualised and designed the study. A. J. N. researched the data with assistance from B. S., M. J. N. and A. G. J. A. J. N. and L. R. conducted data cleaning. A. J. N. analysed the data with assistance from L. R. R., R. A., B. S. and A. G. J. All authors offered advice on the study design, analysis and interpretation of the results. A. J. N. drafted the paper, which was critically revised by all authors. All authors approved the final manuscript.

## FUNDING INFORMATION

This study was funded by a National Institute for Health and Care Research (NIHR) (grant no. NIHR156184) Global Health Group award (17/63/131) and was supported by the NIHR Exeter Biomedical Research Centre. The views expressed are those of the authors and not necessarily those of the NIHR or the Department of Health and Social Care.

## CONFLICT OF INTEREST STATEMENT

The authors declare that they have no known competing interests.

## ETHICS STATEMENT

The study was approved by the UVRI REC (UVRI‐121/2019) and the Uganda National Council of Science and Technology (HS 2588).

## Supporting information


Table S1.

## Data Availability

Data analysed in this study are not available for public use but to researchers upon reasonable request from the corresponding author.
