# Supplementary material for: The impact of prolonged walking on fasting plasma glucose in type 2 diabetes: A randomised controlled crossover study
Source: Diabet Med. 2024 Nov 9;42(3):e15468. doi: 10.1111/dme.15468 (PMC11823290; doi:10.1111/dme.15468)
Supplement: Supplementary file 1 — Table S1. [file DME-42-e15468-s001.docx]

**Table S1: Baseline characteristics baseline variables summarized by sequence (n = 45)**

| Variables | Median (IQR), n (%) | | |
| --- | --- | --- | --- |
|  | **Overall** | **Sequence A**  **(n = 20)** | **Sequence B**  **(n = 25)** |
| Age | 51 (46, 56) | 47.5 (43, 55) | 52 (49, 56) |
| Female | 21 (46.7) | **11 (55)** | **10 (40)** |
| Diabetes duration, years | 4 (2.0, 7.0) | **5.5 (3.0, 7.5)** | **3.0 (1.0, 6.0)** |
| Treatment |  |  |  |
| Metformin (+/-diet only)^a^ | 07 (15.6) | **3.0 (15.0)** | **4 (16.0)** |
| SU (+/- metformin)^b^ | 35 (77.8) | **17 (85.0)** | **18 (72.0)** |
| Other diabetes drugs ^c^ | 03 (06.6) |  | **03 (12.0)** |
| BMI, kg/m^2^ | 26.7 (24.0, 29.7) | **26.7 (22.0, 30.2)** | **26.7 (24.0, 28.7)** |
| Fasting glucose, mmol/L | 7.9 (5.5, 10.0) | **9.4 (5.9, 10.7)** | **7.1 (5.5, 8.6)** |
| HbA1c, mmol/mol | 66 (46, 82) | **71 (49, 83)** | **64 (42, 81)** |
| HbA1c, % | 8.2 (6.4, 9.6) | **8.7 (6.7, 9.8)** | **8.2 (6.0, 9.3)** |
| C-peptide, pmol/L | 1310 (878, 2030) | **1950 (1040, 2670)** | **1130 (865, 1640)** |

**Legend:** Categorical data is presented as frequency (%), continuous data as median (IQR). BMI: Body mass index. a: metformin and diet only (only 1 patient was on diet alone without pharmacological treatment), b: Sulphonylurea with or without metformin, c: Metformin with thiazolidinedione or dipeptidyl peptidase-4 (DPP-4) inhibitor. Sequence A consisted of a walk on the treadmill, followed by a resting visit. In contrast, Sequence B began with a resting visit, which was then followed by the treadmill session.
